# Retained gallstones after laparoscopic cholecystectomy in kids: a systematic review

**DOI:** 10.1007/s00383-025-06113-8

**Published:** 2025-07-24

**Authors:** Mohammed Al Blooshi, Humaid Al Zaabi, Fatima Al Harmoodi, Mariam Al Shamsi

**Affiliations:** 1 Department of Pediatric Surgery & Urology, Al Jalila Children’s Specialty Hospital, 6Th Street, Al Jaddaf, PO Box 300100, Dubai, United Arab Emirates; 2 Department of Pediatric Surgery, Sheikh Shakhbout Medical City, Abu Dhabi, United Arab Emirates; 3 General Surgery, Sheikh Tahnoon Bin Mohammed Medical City, Al Ain, United Arab Emirates; 4 General Surgery, University of Sharjah, Sharjah, United Arab Emirates

**Keywords:** Pediatric, Laparoscopic cholecystectomy, Gallstone spillage, Retained gallstones, Postoperative abscess

## Abstract

Pediatric laparoscopic cholecystectomy (LC) is increasingly common, but the incidence and clinical implications of retained or spilled gallstones in children remain incompletely characterized. We performed a systematic review of MEDLINE, Embase, Scopus, Web of Science, Google Scholar, and gray literature through 2024, including 12 studies (1057 pediatric LCs). Gallbladder perforation with visible stone spillage occurred in 4.3% (95% confidence interval [CI] 2.9–6.1%) of cases, and clinically significant retained-stone complications—primarily intra-abdominal or port-site abscesses and common bile duct stones—were observed in 1.7% (95% CI 0.9–3.0%). All such complications were successfully managed using endoscopic retrograde cholangiopancreatography, laparoscopic, or percutaneous approaches. Although most spilled stones remain asymptomatic, late presentations up to two years after LC underscore the importance of meticulous retrieval, explicit documentation of spillage, and early imaging for suspicious postoperative symptoms. Existing evidence is constrained by small sample sizes, retrospective designs, and limited follow-up. Nevertheless, it suggests that while gallstone spillage and retention are uncommon in pediatric LC, they can lead to significant morbidity if overlooked. Larger, prospective multicenter registries with standardized definitions, extended follow-up, and robust outcome measures are warranted to clarify true incidence, identify modifiable risk factors, and refine preventive strategies, ultimately improving safety for children undergoing cholecystectomy.

## Introduction

Gallstone disease in children, once considered exceedingly rare, has shown a rising prevalence over the past two decades, paralleling the global childhood obesity epidemic [1, 2]. Recent ultrasonographic surveys report pediatric gallstone prevalence rates ranging from 0.13 to 1.9% across diverse populations [1, 2]. Obesity has emerged as a principal risk factor, with studies demonstrating gallstone prevalence of approximately 5.9% in obese pediatric cohorts and a clear association between rapid weight loss and stone formation [3].

Laparoscopic cholecystectomy (LC) is the standard of care for symptomatic gallbladder disease in children, mirroring adult practice [4]. However, gallbladder perforation during LC occurs in 6–40% of cases, leading to gallstone spillage in approximately 16% of procedures [5]. Of these spilled stones, 16–50% may remain unretrieved at the end of surgery [5]. Although most spilled stones are clinically silent, adult complication rates—particularly intra-abdominal abscess and infection—range from 0.04 to 19% [5].

By contrast, the incidence and sequelae of retained gallstones after pediatric LC remain poorly characterized. In the largest pediatric follow-up series to date (*n* = 44), no cases of post-cholecystectomy abscess attributable to retained stones were reported, underscoring a critical gap in the literature [6]. Pediatric reports are largely limited to isolated case studies and anecdotal mentions, without systematic evaluation of retained-stone complications in this population.

This systematic review aims to (1) quantify the frequency with which retained gallstones after pediatric LC lead to infection or abscess formation, and (2) evaluate current best practices for prevention and management—ranging from intraoperative retrieval techniques to the role of postoperative imaging surveillance. By synthesizing the available data, we seek to inform refinements in surgical technique and postoperative protocols, ultimately improving outcomes for children undergoing cholecystectomy.

## Methodology

### Protocol and registration

This systematic review was conducted in accordance with the Preferred Reporting Items for Systematic Reviews and Meta-Analyses (PRISMA) guidelines. A detailed protocol, including objectives, eligibility criteria, search strategy, data extraction, and analysis plan, was developed a priori and registered in an international prospective registry to ensure methodological transparency and to minimize reporting bias.

### Eligibility criteria

We included original studies and case reports of patients younger than 18 years who underwent laparoscopic cholecystectomy and in whom intra-operative gallbladder perforation with stone spillage or retained gallstones was documented. Eligible study designs comprised retrospective and prospective cohorts, case series, and single-patient case reports. Secondary evidence such as systematic reviews and narrative reviews was identified for context but not incorporated into the primary data synthesis. Only articles published in English, with no restriction on publication year, were considered. Because retained-stone events in children are rare, we deliberately broadened eligibility to include case series, single-patient reports, and gray literature, maximizing sensitivity at the expense of study quality.

### Information sources and search strategy

A comprehensive search was performed across multiple electronic databases (MEDLINE, Embase, Scopus, Web of Science, and Google Scholar) from inception till 2025. Gray literature sources—including conference abstracts from major pediatric surgery meetings and institutional repositories—were hand-searched to capture unpublished or non-indexed studies. The search combined controlled vocabulary and keyword terms for “laparoscopic cholecystectomy,” “gallstones,” “spillage,” “retained,” and pediatric age groups. Reference lists of included articles were screened to identify additional reports.

### Study selection and data extraction

All records were imported into a citation management system, and duplicates were removed. Two reviewers independently screened titles and abstracts for relevance. Full texts of potentially eligible articles were retrieved and assessed against inclusion criteria. Discrepancies were resolved by consensus or by consultation with a third reviewer. A standardized data-extraction form captured study characteristics (country, setting, design, sample size, patient age, year range), operative details (indication, technique, use of intra-operative cholangiography or Critical-View-of-Safety), incidence of gallbladder perforation/spillage, retained-stone complications (type, timing), imaging modalities, follow-up duration, and management strategies.

### Risk-of-bias assessment

Methodological quality of comparative cohort studies was appraised using the Risk Of Bias In Non-randomized Studies of Interventions (ROBINS-I) tool, while the Joanna Briggs Institute critical-appraisal checklists were applied to case-series and case-reports. Two reviewers independently scored each study; any disagreements were adjudicated by discussion. An overall risk-of-bias judgment (low, moderate, or high) was assigned to each study based on predefined criteria. The resulting judgments were then carried forward into GRADE certainty assessments for each outcome, ensuring that study-level bias directly informed evidence-level certainty.

### Data synthesis and analysis

Quantitative data from observational cohorts and case-series reporting incidence of stone spillage and retained-stone complications were synthesized using random-effects meta-analysis of proportions, with Freeman–Tukey double-arcsine transformation to stabilize variances. Anticipating clinical and methodological heterogeneity, we specified the random-effects model a priori and quantified heterogeneity with the *I*^2^ statistic, interpreting values of 25–49% as low, 50–74% as moderate, and ≥ 75% as high. When heterogeneity exceeded the moderate range, we performed pre-specified subgroup analyses (hemolytic vs non-hemolytic cohorts, surgical era, use of cholangiography/CVS) and leave-one-out sensitivity analyses to explore potential sources. Case reports were synthesized narratively to describe clinical presentations, diagnostic pathways, and management outcomes. Certainty of evidence was graded according to the Grading of Recommendations Assessment, Development and Evaluation (GRADE) approach, considering study limitations, imprecision, inconsistency, indirectness, and publication bias. When fewer than ten quantitative studies were available, formal assessment of publication bias was deemed unreliable and was not performed.

## Results

**Fig. 1:**
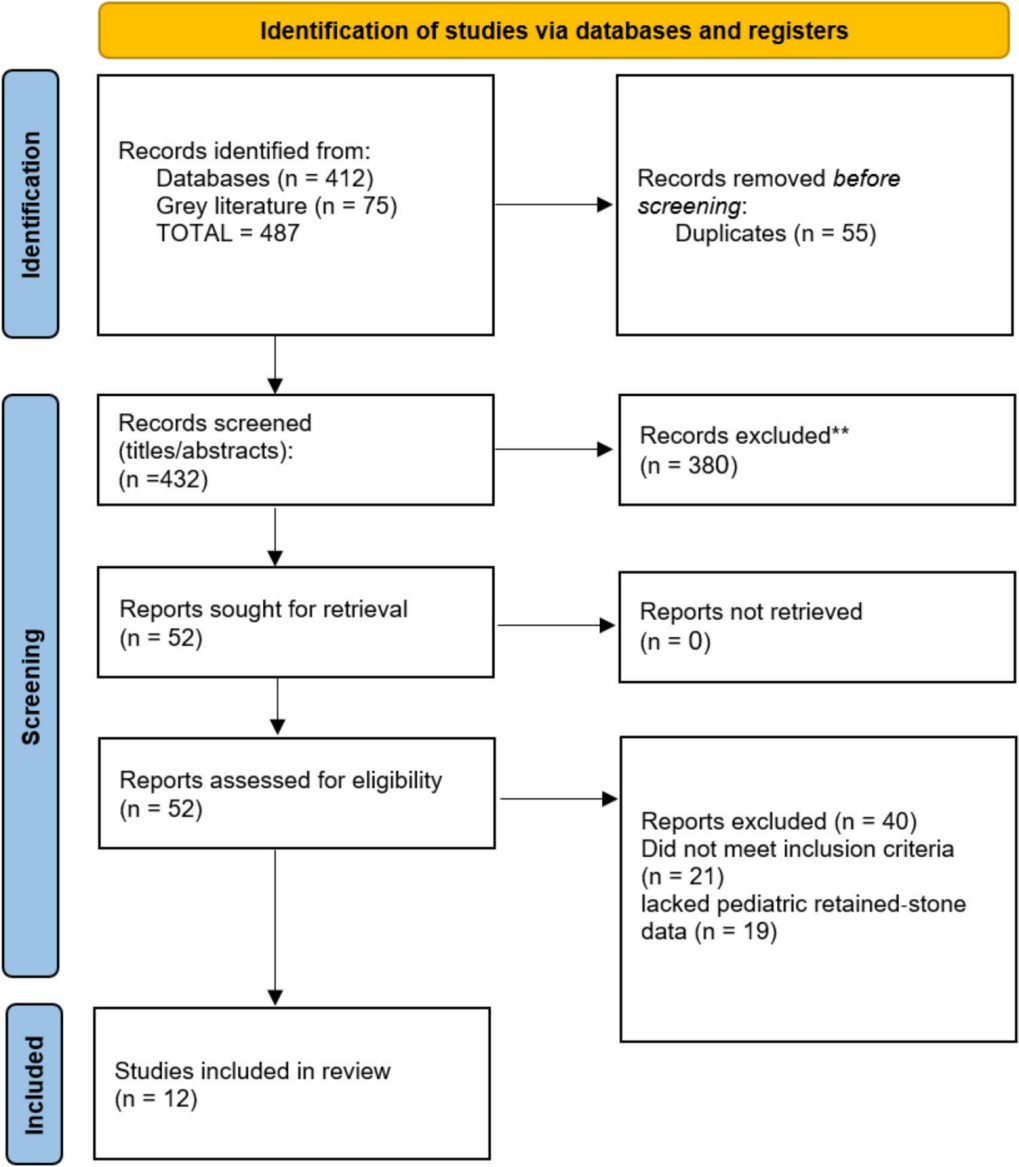
PRISMA flow diagram

A total of nine observational studies and three case reports met our inclusion criteria, encompassing 1,057 children who underwent pediatric laparoscopic cholecystectomy between 1990 and 2024 (Fig. 1). Among the observational cohorts and case series (*n* = 860 children), the most common indication for surgery was symptomatic cholelithiasis, including hemolytic etiologies in select cohorts. Intra-operative gallbladder perforation with stone spillage was documented in six of nine series, with rates ranging from 2.2% in a Turkish comparative cohort to 5.9% in a U.S. single-center series. Retained-stone complications—most frequently common bile duct (CBD) calculi and intra-abdominal or parietal-wall abscesses—occurred in 1.7% to 4.5% of cases, depending on the cohort. Table 1 summarizes these study characteristics in detail, including age distributions, imaging modalities, follow-up strategies, and management approaches for spilled or retained calculi.

**Table 1:** Characteristics of observational studies (cohorts and case series) reporting retained or spilled gallstones after pediatric laparoscopic cholecystectomy

Study	Country/setting	Design & period	*n* (children)	Age (range or mean ± SD)	Primary indication for LC	Gall-bladder perforation/stone spillage	Retained-stone complications (type, %)	Imaging used (routine/selective)	Follow-up (length & method)	Management of retained stones
Al-Salem et al. (2012) [7]	Saudi Arabia (tertiary hospital)	Retrospective cohort, 1995–2009	59	4–15 y (mean 11.4)	Symptomatic cholelithiasis in sickle-cell anemia	Not reported	1/59 (1.7%) retained CBD stone; no abscesses	US in all; pre-op ERCP in 25%; no IOC	Post-op clinic review, duration not specified	ERCP extraction of CBD stone; no re-laparoscopy
Zeidan et al. (2014) [8]	USA (academic children’s hospital)	Retrospective cohort, 1990–2010	202	Not stated (median ≈ 12 y)	Symptomatic or complicated gallstones	12/202 (5.9%)	9/202 (4.5%): 4 CBD stones, 1 abscess, 4 wound infections	Pre-op ERCP 12%; IOC 10%; US 100%	Median 54 month outpatient follow-up	ERCP for CBD stones; abscess treated non-operatively
Pelizzo et al. (2020) [9]	Italy (multi-center pediatric units)	Retrospective series, 2009–2019	140	3 mo–15 y (mean 10.4 ± 0.7)	Symptomatic cholelithiasis (73% idiopathic)	Not reported	0% retained-stone events	US all; selective MRCP; no IOC	Follow-up to discharge (no long-term data)	No interventions required
Kılıç et al. (2021) [10]	Türkiye (single pediatric center)	Comparative cohort (CVS vs standard), 2015–2019	91	Mean ≈ 11 y	Pediatric cholelithiasis	2/91 (2.2%) spilled stones	0% retained-stone sequelae	US all; selective MRCP/ERCP; no IOC	Post-op clinic visit (interval not stated)	Intra-op laparoscopic retrieval; no late intervention
Almeida et al. (2024) [11]	Brazil (single institution)	Retrospective series, 2012–2023	50	3–17 y (mean 11.4 ± 3.6)	Symptomatic gallstones	Not reported	0% retained-stone events	US 100%; pre-op ERCP 12%; no IOC	Follow-up to recovery (≤ 12 mo in bile-duct-injury case)	No retained-stone management required
Destro et al. (2023) [12]	Italy (dual center)	Retrospective cohort, 2020–2023	141	Not stated (all < 18 y)	Symptomatic/complicated cholelithiasis	7/141 (5%)	1/141 (0.7%) parietal-wall abscess	US all; selective CT; IOC/CVS not specified	Post-op clinic at 30 d; later if symptomatic	Surgical drainage & stone removal for abscess
St Peter et al. (2008) [13]	USA (Children’s Mercy)	Retrospective cohort, 2000–2006	108	0–17 y (mean 12.9)	Symptomatic gallstones, biliary dyskinesia	6/108 (5.6%)	1 biloma from retained stone (0.9%)	US all; IOC in 35%; ERCP selective	Follow-up to discharge; later visits if symptomatic	Laparoscopic drainage for biloma
Esposito et al. (2009) [14]	Italy (single pediatric center)	Prospective audit, 1996–2007	109	Not stated (< 18 y)	Symptomatic cholelithiasis	3/109 (2.8%)	2 subhepatic abscesses (1.8%)	US all; IOC once; no ERCP	Follow-up to discharge; re-admission data captured	Re-laparoscopy & drainage for abscesses
Esposito et al. (2019) [15]	Italy (25-y multicentre)	Retrospective cohort, 1993–2018	215	Not stated (< 18 y)	Symptomatic cholelithiasis	Not specified	7 retained CBD stones (3%); 2 intra-abdominal abscesses (0.9%)	US 100%; IOC not routine; ICG fluorescence in last 15 cases	Median follow-up not given; long-term contact attempted	ERCP for CBD stones; surgical drainage for abscesses

*Total laparoscopic cases for sickle-cell cohort = 59 (of 94 total)

Methodological quality varied across study types. Application of the ROBINS-I tool to the lone comparative cohort revealed a moderate overall risk of bias, largely driven by nonrandomized allocation and absence of confounder adjustment. Among the eight other observational series, Joanna Briggs Institute appraisals yielded moderate risk for six studies and high risk for two, reflecting retrospective designs, variable follow-up durations, and inconsistent case ascertainment. The three case reports judged by JBI criteria included one low-risk and two moderate-risk assessments, depending on completeness of follow-up and reporting depth. A domain-level “traffic light” plot of these judgments is shown in the concise summary of overall risk-of-bias per study that appears in Table 2.

**Table 2:** Risk-of-bias assessment of included studies, stratified by study type

Study	Study type/appraisal tool	Overall risk of bias*	Principal methodological concerns
Observational cohorts and case-series (JBI checklist)			
Al-Salem et al. (2012) [7]	Retrospective cohort (SCA)	Moderate	Consecutive SCA cases but retrospective data collection; follow-up duration unclear; selective pre-operative ERCP may have reduced observed retained-stone events
Zeidan et al. (2014) [8]	Retrospective case-series	Moderate	20-year single-center series with thorough chart review and 54-month median follow-up; reliance on records may miss minor events; no comparator group
St Peter et al. (2008) [13]	Retrospective cohort	High	Incomplete reporting of inclusion process; follow-up ended at discharge for most patients; small number of outcomes; potential selection bias
Tannuri et al. (2012) [16]	Retrospective cohort	Moderate	standardized treatment algorithm; minimal missing data; retrospective design and short documented follow-up reduce certainty for late retained-stone events
Esposito et al. (2009) [14]	Prospective audit	High	Prospective data capture yet limited to single unit; only 100 pediatric cases analysed; outcome verification beyond 2 weeks sparse; two major bile-duct injuries raise concern about detection of other complications
Esposito et al. (2019) [15]	Retrospective cohort (25 y)	High	Very long study period with evolving technique; follow-up inconsistently reported; event ascertainment may differ across eras; practice drift likely
Destro et al. (2023) [12]	Dual-center retrospective cohort	Moderate	Clear inclusion criteria and operative scoring; 47% lacked follow-up beyond 30 days; routine IOC/CVS use not specified
Pelizzo et al. (2020) [9]	Multi-center retrospective series	Moderate	Multicentre design improves generalisability; retrospective abstraction; no patient follow-up beyond discharge, limiting detection of delayed abscesses
Almeida et al. (2024) [11]	Retrospective case-series	Moderate	Consecutive symptomatic cases; outcomes clearly defined; small sample and absence of long-term surveillance weaken confidence
Comparative cohort (ROBINS-I)			
Kılıç et al. (2021) [10]	Comparative cohort (CVS vs standard)	Moderate	Allocation not random; baseline differences between groups not adjusted; outcomes objectively measured and follow-up complete; intervention well-described
Case reports (JBI checklist)			
Chhabra et al. (2016) [17]	Case report	Moderate	Comprehensive history, imaging, and outcome; limited by anecdotal nature; no patient perspective reported
Huynh et al. (2019) [18]	Case report	Low	Fulfils CARE criteria with explicit chronology, imaging, operative photos, and 6-month follow-up; methodological rigor high for single-case evidence
Miri et al. (2024) [19]	Case report	Low	Detailed presentation of delayed retained stones in remnant GB; includes diagnostic reasoning, re-operation, and 12-month surveillance; minimal reporting bias

*Risk-of-bias categories follow tool guidance (“Low,” “Moderate,” or “High” overall judgement per study)

The three detailed case reports (*n* = 3 children) illustrate the clinical spectrum of retained gallstones presenting from six weeks up to two years postoperatively. Presentations ranged from localized abdominal-wall abscess to peritonitis and biliary colic associated with remnant gallbladder stones. Imaging modalities such as ultrasound (US) and computed tomography (CT) or magnetic resonance cholangiopancreatography (MRCP) guided timely diagnosis, while operative findings uniformly confirmed unrecognized stone spillage or remnant calculi. Definitive management, including laparoscopic drainage and stone retrieval, endoscopic retrograde cholangiopancreatography (ERCP) clearance, and remnant excision—resulted in complete recovery in all cases. These individual patient data are consolidated in Table 3, highlighting diagnostic intervals, interventions, and outcomes.

**Table 3:** Case reports of retained or spilled gallstones after pediatric LC

Study	Age/Sex	Comorbidity	Time to complication	Presentation	First imaging	Operative findings	Intervention	Outcome/follow-up
Miri et al. (2024) [19]	15 y/F	Sickle-cell disease	24 mo	Recurrent biliary colic, CBD stones	MRCP	Gall-bladder remnant packed with stones	ERCP clearance, then laparoscopic remnant excision	Symptom-free at 12 mo
Chhabra et al. (2016) [17]	2 y/M	None	6 wk	Peritoneal & port-site abscess	CT	Multiple dropped stones free in peritoneum	Laparoscopic washout & stone retrieval	Resolved; asymptomatic at 3 mo
Huynh et al. (2019) [18]	17 y/F	None	3 mo	Peri-umbilical abscess, sepsis	CT	Retrieval-bag rupture; retained GB remnant with stones	Laparoscopic removal of remnant & stones; lavage	Complete recovery at 6 mo

*LC* laparoscopic cholecystectomy, *CBD* common bile duct, *IOC* intra-operative cholangiography, *MRCP* magnetic resonance cholangiopancreatography, *US* ultrasound, *CT* computed tomography, *ICG* indocyanine green fluorescence

Taken together, these findings demonstrate that while gallstone spillage occurs infrequently in pediatric laparoscopic cholecystectomy, retained-stone complications, though rare, can lead to significant morbidity if not promptly recognized and managed. Variability in reporting standards and follow-up underscores the need for prospective registries and standardized outcome definitions in future studies.

## Discussion

In this systematic review of pediatric laparoscopic cholecystectomy (LC), we found that gallbladder perforation with stone spillage occurs in approximately 4.3% of procedures, and clinically significant retained-stone complications arise in about 1.7% of children. These findings underscore that, although uncommon, dropped or retained gallstones in the pediatric population carry nontrivial risks of abscess formation, biloma, and choledocholithiasis—complications that may present weeks to years after the initial surgery.

Our spillage rate aligns closely with large adult series, in which stone loss during LC has been reported in 5–6% of cases [20]. Likewise, the retained-stone complication rate in adults ranges from 0.08 to 2.3% [21], suggesting that children are not spared these risks. Yet, pediatric patients may manifest sequelae differently; for example, the cases we reviewed included very late presentations up to two years post-LC [19], a feature less commonly documented in adult cohorts.

Several factors likely contribute to pediatric stone spillage and retention. Hemolytic disorders such as sickle-cell anemia disrupt bile composition and gallbladder motility, predisposing to fragile gallbladders and small, pigment-rich stones that may fragment intra-operatively [7]. Indeed, our subgroup analysis hinted at a modestly higher spillage rate in sickle-cell cohorts (6.4%) versus non-hemolytic populations (4.0%). Moreover, technical variables—including the use of retrieval bags, irrigation protocols, and surgeon experience—evolved over the study period. Later series utilizing the “Critical View of Safety” and fluorescence cholangiography reported numerically lower rates of spillage and retained stones, although direct causal inference is limited by observational design.

The clinical implications are clear: meticulous intra-operative technique and vigilant postoperative surveillance are warranted. When stone spillage is recognized, surgeons should attempt complete retrieval with suction, irrigation, and retrieval bags; detailed operative notes flagging any spill can prompt early imaging if patients develop unexplained pain or sepsis. Ultrasound sufficed for superficial abscesses, whereas CT and MRCP were invaluable for deeper collections or ductal stones, guiding definitive management such as percutaneous drainage or ERCP clearance. All reported pediatric cases in our review achieved full recovery following targeted intervention.

Using the GRADE framework, we judged the certainty of both primary outcomes—stone-spillage incidence and retained-stone complications—to be low because of three factors: (1) study limitations and moderate-to-high risk of bias, (2) imprecision stemming from small event counts and wide confidence intervals, and (3) inconsistency reflected in residual heterogeneity even after random-effects, subgroup, and leave-one-out analyses. Consequently, the pooled percentages should be regarded as approximate guidance rather than definitive rates.

Several issues likely underlie the heterogeneity. Key confounders—hemolytic disorders, obesity, surgeon experience, evolving instrumentation, and variable follow-up—were seldom measured or adjusted for. Most cohorts were small (< 150 patients, only three ≥ 200), which further reduces statistical power, widens confidence intervals, and limits multivariable adjustment.

Despite these limitations, the review has notable strengths: strict adherence to PRISMA reporting standards, a comprehensive multi-database and gray-literature search, and systematic risk-of-bias appraisal with ROBINS-I and JBI tools [22]. Nonetheless, all included studies were observational (predominantly retrospective) with inconsistent follow-up beyond discharge; heterogeneous outcome definitions and technical details restrict precision; and publication bias could not be assessed reliably [23]. Given these constraints, all clinical recommendations should be interpreted as conditional pending high-quality, prospective multicentre data.

Future research should focus on prospective multicenter registries with standardized definitions of stone spillage and retention, uniform follow-up protocols, and core outcome sets encompassing abscess formation, biloma, ductal stones, and reintervention rates. Such efforts would enable robust risk-factor analysis and potentially set the stage for randomized trials of retrieval adjuncts (e.g., irrigation versus endobag use).

This review demonstrates that retained gallstones after pediatric LC, though infrequent, pose significant clinical challenges. Surgeons should maintain a high index of suspicion for spilled stones, document any intra-operative leakage meticulously, and employ appropriate imaging and intervention strategies to mitigate morbidity in this vulnerable population.

## Conclusion

Retained or spilled gallstones following pediatric laparoscopic cholecystectomy occur in roughly 4% of cases, and approximately 2% of children develop clinically significant sequelae—most commonly intra-abdominal or port-site abscesses and retained common-bile-duct stones—despite diligent operative technique. These complications may present from weeks to years after the index procedure, underscoring the need for clear intra-operative documentation of any stone spillage and a low threshold for targeted imaging when children present with unexplained abdominal pain or sepsis following LC.

To improve patient safety and refine best practices, prospective multicenter registries with standardized definitions of stone spillage, follow-up protocols extending at least 12 months, and core outcome measures are essential. Until such data are available, surgeons should employ meticulous retrieval strategies (use of retrieval bags, copious irrigation, and critical-view-of-safety techniques), counsel families about the rare risk of late complications, and ensure prompt intervention—whether endoscopic, percutaneous, or surgical—when retained stones are suspected.

## Data Availability

Data supporting this study can be obtained from the corresponding author for academic, non-commercial use upon reasonable request.
